# Supplementary material for: Tiltable objective microscope visualizes selectivity for head motion direction and dynamics in zebrafish vestibular system
Source: Nat Commun. 2022 Dec 21;13:7622. doi: 10.1038/s41467-022-35190-9 (PMC9772181; doi:10.1038/s41467-022-35190-9)
Supplement: Supplementary file 1 — Supplementary Information [file 41467_2022_35190_MOESM1_ESM.pdf]

Supplementary Information

Tiltable objective microscope visualizes selectivity for head motion direction and dynamics in zebrafish vestibular system

Masashi Tanimoto,[1,2,*] Ikuko Watakabe,[1,2] Shin-ichi Higashijima[1,2,*]

[1]Division of Behavioral Neurobiology, National Institute for Basic Biology, Okazaki, Aichi 444-8787, Japan

[2]Neuronal Networks Research Group, Exploratory Research Center on Life and Living Systems, Okazaki, Aichi 444-8787, Japan

[*]Correspondence: tanimoto@nibb.ac.jp; shigashi@nibb.ac.jp

Supplementary Table 1. Maximum amplitude of static tilt-evoked fluorescent intensity ratio changes in HCs in 6 utricles.

HC functional group	Mean (%)	Standard error of the mean (%)
Nose-down preferred, biphasic	89.5	19.7
Nose-down preferred, monophasic	91.3	9.8
Tail-down preferred, biphasic	74.3	5.6
Tail-down preferred, monophasic	71.4	10.0
Lateral-down preferred, biphasic	82.9	7.5
Lateral-down preferred, monophasic	65.2	11.3
Medial-down preferred, biphasic	72.0	2.5
Medial-down preferred, monophasic	45.7	4.6

Supplementary Table 2. List of key optomechanical components.

Optomechanical components	Manufacturers	Product Identifier
Fluorescent beads	ThermoFisher Scientific	T7282
Rotation stage unit: Motorized rotation stage	ThorLabs	DDR100/M
Rotation stage unit: Motor controller	ThorLabs	BBD201
Rotation stage unit: Stage adaptor plate	ThorLabs	NR360SP8
Objective lens unit: Kinematic mirror mount	ThorLabs	KCB1EC/M
Objective lens unit: Elliptical mirror	ThorLabs	PFE10-P01
Objective lens unit: Z translation lens positioner	ThorLabs	SM1Z
Objective lens unit: Objective lens (20×, N.A. 0.8)	Olympus	UPLXAPO20X
Objective lens unit: Objective lens (40×, N.A. 0.8)	Olympus	LUMPLFLN40XW
Objective lens unit: Cage mount	OptoSigma	C30-CMP-H32
Objective lens unit: XY translation mount	ThorLabs	CXY1
Objective lens unit: Rotation mount	ThorLabs	CRM05
Tube lens unit: Camera port with a tube lens (f = 200 mm)	ThorLabs	WFA4100 (tube lens: TTL200)
Tube lens unit: Cage adaptor plate	ThorLabs	CSA1003
Tube lens unit: Cage adaptor plate	ThorLabs	LCP02/M
Confocal scanner unit	Yokogawa	CSU-X1
Confocal scanner unit: Dichroic mirror	Semrock	Di01-T405/488/561-13x15x0.5
Image splitting optics: W-VIEW GEMINI	Hamamatsu	A12801-01
Image splitting optics: Dichroic mirror for image splitting optics	Semrock	FF560-FDi01-25x36
Image splitting optics: Emission filter	Semrock	FF01-512/25-25
Image splitting optics: Emission filter	Semrock	FF02-617/73-25
Digital sCMOS camera ORCA-Flash 4.0 V3	Hamamatsu	C13440-20CU
Laser	Coherent	Sapphire 488-50
Specimen chamber: FEP sheet: thickness 50 μm	Flonchemical	NR0538-002
Accelerometer	MicroStone	MA3-04AD
Digitizer	Molecular Devices	Digidata 1440A

Supplementary Table 3. List of key software and algorithms.

Software and algorithms	Manufacturers/Source	Uniform Resource Locator
HCImage Live	Hamamatsu Photonics	https://hcimage.com/hcimage-overview/hcimage-live/
Kinesis	ThorLabs	https://www.thorlabs.com/newgrouppage9.cfm?objectgroup_id=10285
MATLAB	MathWorks	https://www.mathworks.com/products/matlab.html
Image Processing Toolbox	MathWorks	https://www.mathworks.com/products/image.html
dftregistration	Guizar-Sicairos et al., 2008	https://www.mathworks.com/matlabcentral/fileexchange/18401-efficient-subpixel-image-registration-by-cross-correlation
ImageJ	National Institutes of Health	https://imagej.nih.gov/ij/
Python	Python Software Foundation	https://www.python.org/
SciPy	Virtanen et al., 2020	https://scipy.org/
pClamp	Molecular Devices	https://www.moleculardevices.com/products/axon-patch-clamp-system/acquisition-and-analysis-software/pclamp-software-suite#gref

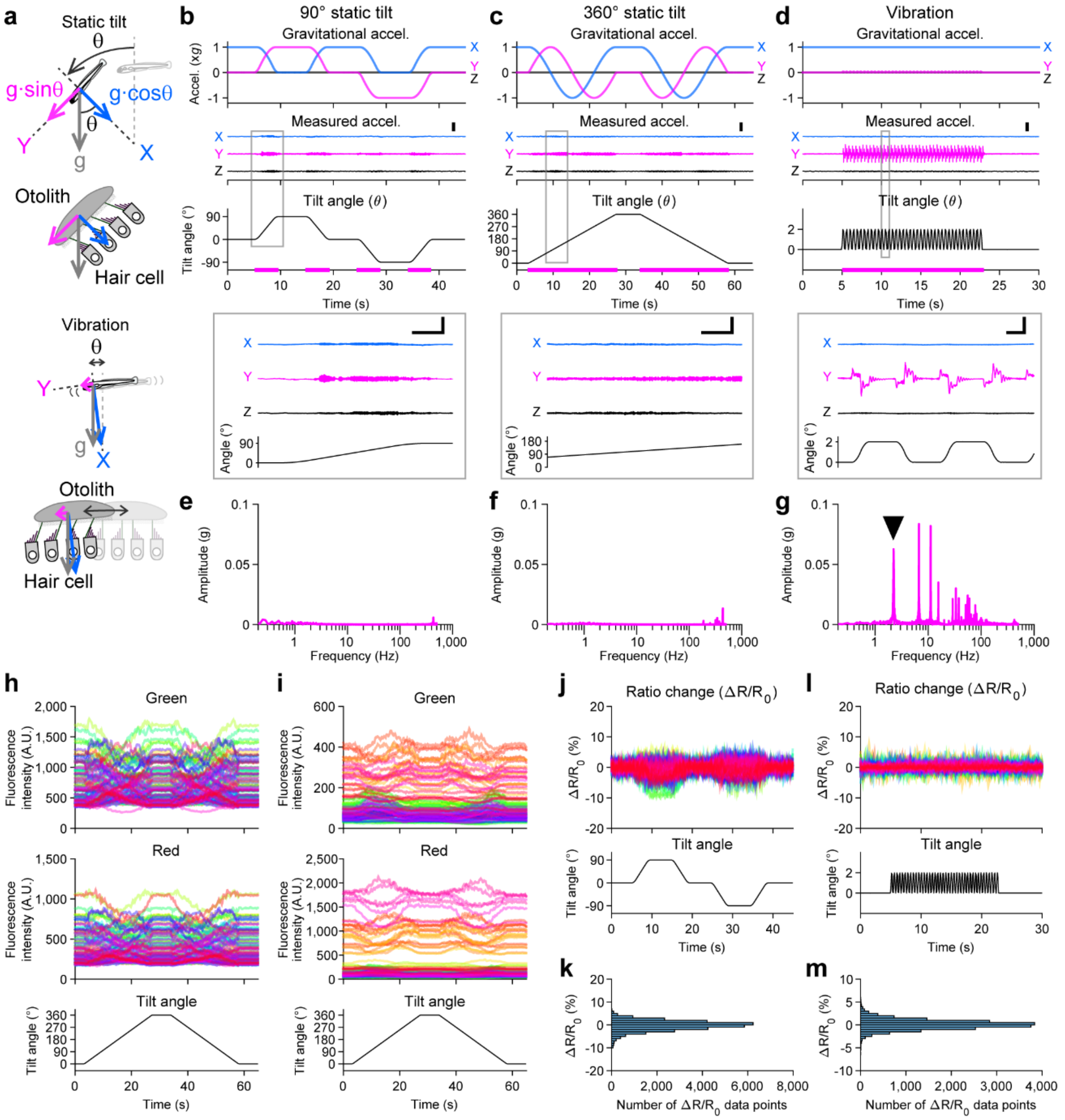

Supplementary Figure 1. Characterization of tiltable objective microscope.

a Schematic showing axes of acceleration in fish and otolith organs during tilt and vibration. Stage rotation with an angle, θ, produces vector components of gravitational acceleration (**g**, gray arrow): $\mathbf{g} \cdot \cos\theta$ (blue arrow) in the centripetal axis (X) and $\mathbf{g} \cdot \sin\theta$ (magenta arrow) in the tangential axis (Y) that is orthogonal to the centripetal axis X. The otolith is much denser than the surrounding tissues. During static tilt, the otolith slides down relative to HCs, which provides a sustained deflection of the hair bundles and stimulates the HCs. During vibration, the otolith

moves less and lags behind the HCs. This relative displacement between the otolith and HCs deflects the hair bundles and stimulates the HCs.

b-d Acceleration during 90° static tilt (**b**), 360° static tilt (**c**), and vibration (**d**) stimulus. Gravitational acceleration simulated by vector components in (**a**) (top). The Z axis is orthogonal to the X and Y axes. Measured inertial acceleration (middle). Time course of tilt angle (bottom). Scale bar: 0.5 g. Close-up view of the rectangle areas (insets). Measured maximum inertial acceleration: 90° tilt: X: 0.047 ± 0.006 g; Y: 0.159 ± 0.016 g; Z: 0.054 ± 0.006 g; 360° tilt: X: 0.030 ± 0.001 g; Y: 0.094 ± 0.003 g; Z: 0.043 ± 0.002 g; vibration: X: 0.026 ± 0.001 g; Y: 0.529 ± 0.010 g; Z: 0.031 ± 0.001 g, mean ± SEM, 5 trials. Vertical bars: 0.5 g. Horizontal bars: 1 s for (**b, c**) and 100 ms for (**e**).

e-g Frequency spectra of acceleration in the Y axis during stage motion (indicated by magenta lines on the time axis in [**b-d**]). 90° static tilt (**e**), 360° static tilt (**f**), and vibration (**g**) stimulus. Single-sided amplitude spectrum is shown. Arrowhead in (**g**) indicates 2.2 Hz, which corresponds to the cycle frequency of angle changes (bottom in [**d**]).

h Time course of fluorescence intensity in beads during 360° static tilt.

i Time course of fluorescence intensity in Kaede-expressing neurons during 360° static tilt.

j Changes in fluorescence intensity ratio ($\Delta R/R_0$) in Kaede-expressing neurons during 90° static tilt.

k Distribution of $\Delta R/R_0$ values during 90° static tilt.

l $\Delta R/R_0$ in Kaede-expressing neurons during vibration stimulus.

m Distribution of $\Delta R/R_0$ values during vibration stimulus.

Source data are provided as a Source Data file.

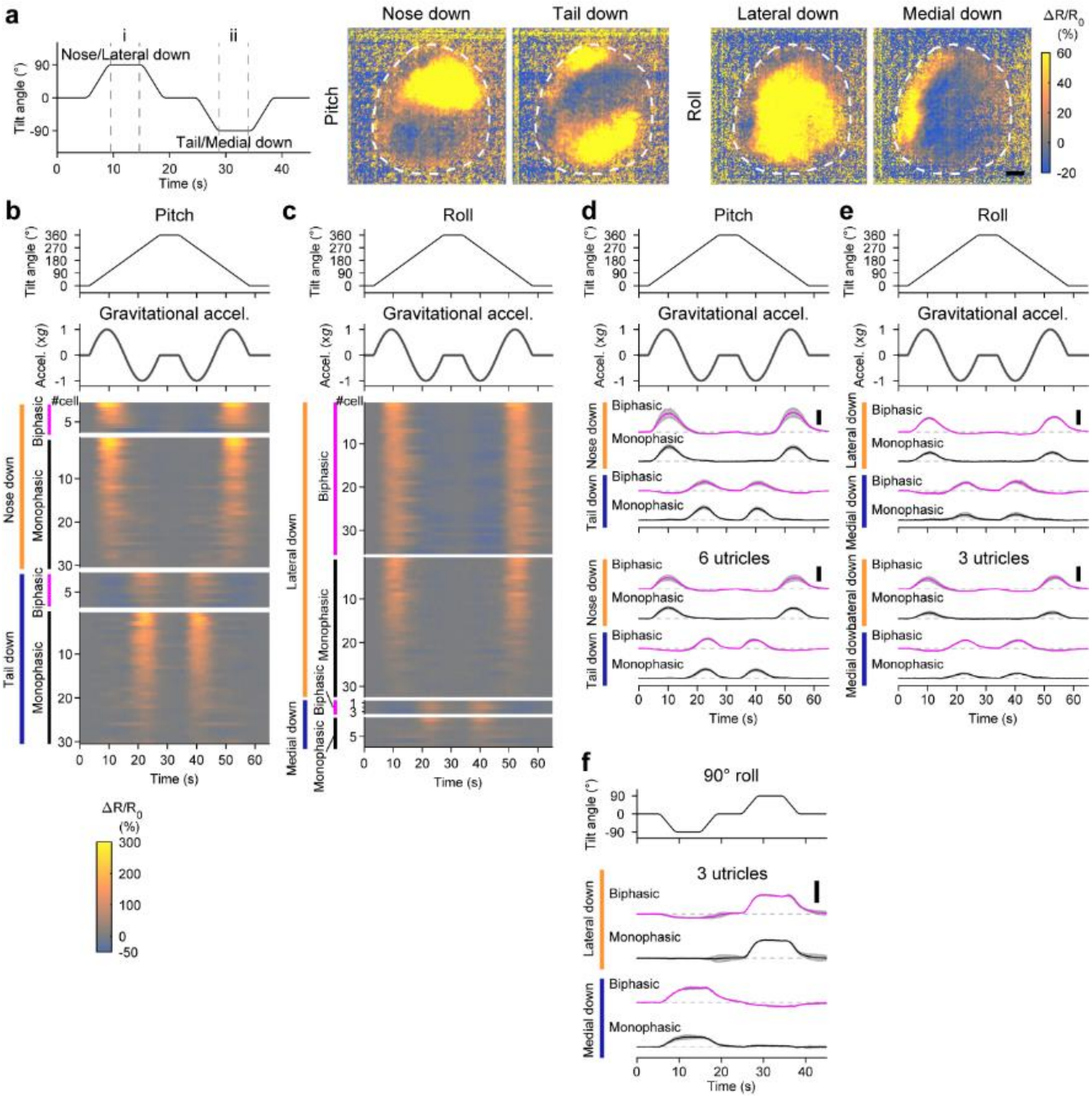

Supplementary Figure 2. Utricular HC responses to static tilt.

a Utricular HC responses to 90° static tilt. Mean $\Delta R/R_0$ during the period "i" and "ii" showing positive (yellow) and negative (blue) responses during nose-down, tail-down, lateral-down, and medial-down tilt. Dashed lines: utricle contours. Color scale range is chosen to show small-amplitude negative responses. Average of 6 utricles. Scale bar: 10 µm.

b, **c** HC responses to 360° static tilt in the pitch (**b**) and roll (**c**) axes. Data from the same utricle shown in Figure **2b**. Tilt angle: 90° and 270° in the pitch axis indicate nose down and tail down, respectively; 90° and 270° in the roll axis indicate lateral down and medial down, respectively (top row). Gravitational acceleration in the tangential direction (Y in Supplementary Fig. **1a**) that

provides hair bundles with shearing force during the tilt (second row). A positive value indicates acceleration to the nose down in the pitch axis and lateral down in the roll axis. $\Delta R/R_0$ responses grouped by preferred tilt direction and biphasic/monophasic response patterns, both of which are determined by responses to 90° tilt (Fig. **2d**). Average of 3 trials. Color bar applies to (**b**) and (**c**).

d, **e** Mean HC responses to 360° tilt in the pitch (**d**) and roll (**e**) axes. Top and second rows are the same as in (**b**, **c**). Mean $\Delta R/R_0$ responses of each functional group shown in (**b**, **c**) (third row). Number of cells in each group: Nose-down preferred group: 7 biphasic cells, 30 monophasic cells; Tail-down preferred group: 8 biphasic cells, 30 monophasic cells; Lateral-down preferred group: 35 biphasic cells, 32 monophasic cells; Medial-down preferred group: 3 biphasic cells, 7 monophasic cells. Mean $\Delta R/R_0$ responses of 6 utricles for pitch and 3 left utricles for roll (bottom row). Deviation (SEM) is shown in gray. Number of cells in each group: Nose-down preferred group: 11 ± 2.9 biphasic cells, 25 ± 4.5 monophasic cells; Tail-down preferred group: 7.3 ± 2.0 biphasic cells, 27 ± 3.0 monophasic cells; Lateral-down preferred group: 34 ± 4.5 biphasic cells, 27 ± 7.3 monophasic cells; Medial-down preferred group: 3.3 ± 0.2 biphasic cells, 9.5 ± 2.1 monophasic cells (mean ± SEM, 6 utricles). Scale bar: 100% $\Delta R/R_0$.

f Mean HC responses to 90° roll tilt in 3 right utricles. Tilt angle: positive value indicates lateral down (top row). Mean $\Delta R/R_0$ responses (bottom). Deviation (SEM) is shown in gray. Scale bar: 100% $\Delta R/R_0$.

Source data are provided as a Source Data file.

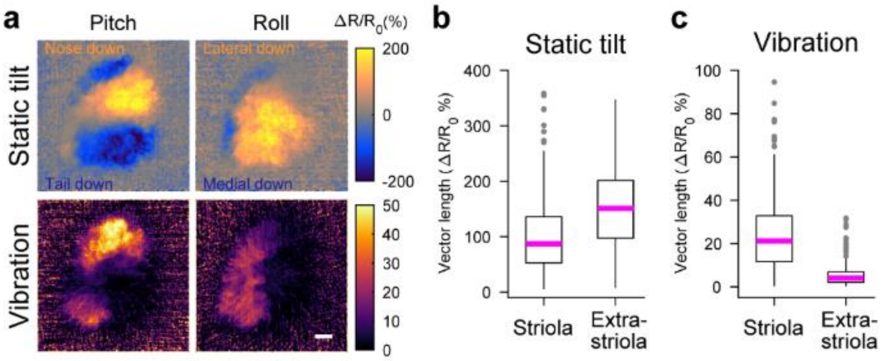

Supplementary Figure 3. Activity map and vector length of static tilt and vibration responses.

a Activity maps showing direction-selective responses to static tilt (top) and vibration (bottom) stimulus in utricular HCs in *Tg(myo6b:jGCaMP7f; s100s-hs:tdTomato)* larvae. Similar to Figure **2c**, tail-down response image subtracted from nose-down response image for the pitch axis (top left). Medial-down response image subtracted from lateral-down response image for the roll axis (top right). Similar to Figure **6a**, changes of mean $\Delta R/R_0$ during the time period "iv" from those during the pre-stimulus period are shown (bottom). Average of 6 utricles. Scale bar: 10 µm. Rostral is to the top. Lateral is to the left.

b Box plots of the $\Delta R/R_0$ response vector length in 90° static tilt responses (Fig. **4c**) compared between striolar and extrastriolar HCs. Magenta bars indicate the median. The top and bottom of the boxes indicate 75th and 25th percentiles, respectively. Top ends of upper whiskers indicate the 99th percentile. Bottom ends of lower whiskers indicate the 1st percentile. Dots indicate outliers above the 99th percentile. Striolar HCs: 297 cells. Extrastriolar HCs: 229 cells. Two-sided Mann–Whitney U test, $p = 9.3 \times$ e-14.

c Box plots of the vector sum length of $\Delta R/R_0$ bars in vibration responses (Fig. **4d**) compared between striolar and extrastriolar HCs. Parameters of box plots and number of cells are the same as in (**b**). Two-sided Mann–Whitney U test, $p = 1.3 \times$ e-54.

Source data are provided as a Source Data file.

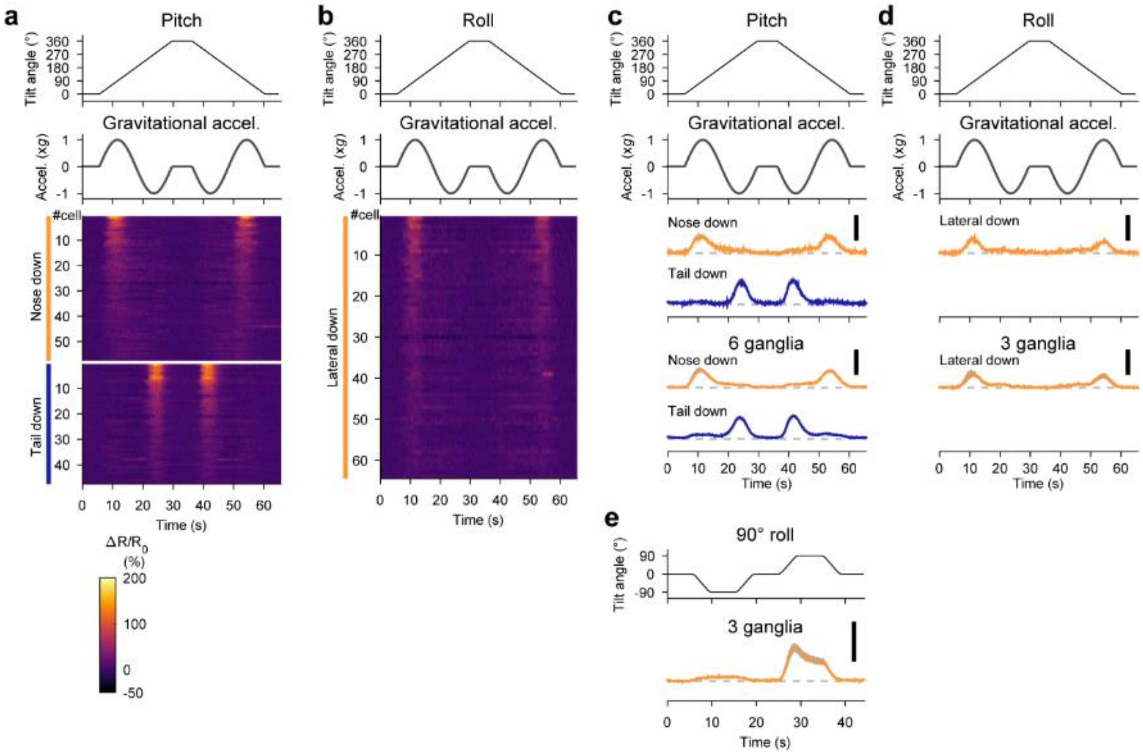

Supplementary Figure 4. VGN responses to static tilt.

a, **b** VGN responses to 360° tilt in pitch (**a**) and roll (**b**) axes. The same VGNs shown in Figure **5b**. Tilt angle time course (top row). Gravitational acceleration vector component that provides HCs with shearing force in the tangential direction (Y in Supplementary Fig. **1a**) during tilt (second row). Heat map showing $\Delta R/R_0$ responses grouped by preferred tilt direction that is determined by responses to 90° tilt. Color bar applies to (**a**) and (**b**).

c, **d** Mean VGN responses to 360° tilt in pitch (**c**) and roll (**d**) axes. Tilt angle time course (top row). Gravitational acceleration vector component in the tangential direction (Y in Supplementary Fig. 1a) during tilt (second row). Mean $\Delta R/R_0$ responses of each VGN group shown in (**a**) and (**b**) (third row). Nose-down preferred: 57 cells; tail-down preferred: 47 cells; lateral-down preferred: 64 cells. Mean $\Delta R/R_0$ responses of 6 ganglia for pitch and 3 left ganglia for roll (bottom row). Deviation (SEM) is shown in gray. Scale bar: 50% $\Delta R/R_0$.

e Mean VGN responses to 90° roll tilt in 3 right ganglia. Tilt angle: a positive value indicates lateral down (top row). Mean $\Delta R/R_0$ responses (bottom). Deviation (SEM) is shown in gray. Scale bar: 50% $\Delta R/R_0$.

Source data are provided as a Source Data file.

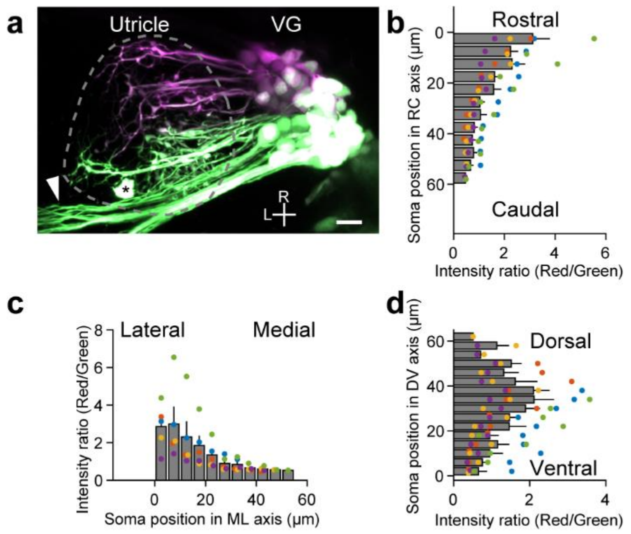

Supplementary Figure 5. Topographic pattern in VGN innervation of utricular HCs.

a Maximum intensity projection image of VGNs expressing Kaede in a *Tg(hspGFF53A; UAS:Kaede)* larva at 5 dpf. Green-Kaede (green) in the peripheral neural processes is photoconverted to red-Kaede (magenta) only in the rostral half in the utricle. The red-Kaede diffuses from the neural processes to VGN somata. Dashed line encloses the utricle. Arrowhead: horizontal canal nerve. Asterisk: ectopic Kaede-expressing cell. R: rostral. L: lateral. Scale bar: 10 μm.

b-**d** Distribution of fluorescence intensity ratio (red/green) in VGN somata in the rostrocaudal (RC) (**b**), mediolateral (ML) (**c**), and dorsoventral (DV) (**d**) axes. Large ratio values indicate a large amount of photoconverted Kaede fluorescence in the somata. Data from 5 utricles/ganglia. Bars indicate mean and whiskers indicate SEM. The positions of the most rostral (**b**), lateral (**c**), or ventral (**d**) VGN are defined as zero. Since the number of labeled VGNs slightly varies across fish in the transgenic line, the number of data points declines in the caudal (**b**), medial (**c**), and dorsal (**d**) ends.

Source data are provided as a Source Data file.
