# Supplementary material for: Tiltable objective microscope visualizes selectivity for head motion direction and dynamics in zebrafish vestibular system
Source: Nat Commun. 2022 Dec 21;13:7622. doi: 10.1038/s41467-022-35190-9 (PMC9772181; doi:10.1038/s41467-022-35190-9)
Supplement: Supplementary file 3 — Description of Additional Supplementary Files [file 41467_2022_35190_MOESM3_ESM.docx]

**Description of Additional Supplementary Files**

**Supplementary Movie 1:** Movement of tiltable objective microscope during 360° static tilt and vibration stimulus. Movement of the objective lens unit on the rotation stage during 360° static tilt and vibration stimulus imaged from the front side of the microscope. ×1 playback speed.

**Supplementary Movie 2:** Raw images, tracking beads position, and registered images. Three sequential movie clips at ×4 playback speed. First clip: sequential raw images of fluorescent beads in green and red channels. Second clip: beads position tracked in color-merged channel. Third clip: registered sequential images.

**Supplementary Movie 3:** Utricular HC responses to 90° static tilt. Two sequential movie clips at ×4 playback speed showing ΔR/R0 utricular HC responses to 90° static tilt. First clip: pitch axis. Second clip: roll axis. Average of 5 trials.

**Supplementary Movie 4:** Utricular HC responses to vibration stimulus. Two sequential movie clips at ×4 playback speed showing ΔR/R0 utricular HC responses to vibration stimulus. First clip: pitch axis. Second clip: roll axis. Average of 8 trials. Baseline ratio image was the mean of ratio image frames ±2.5 s from the beginning of the stimulus onset.

**Supplementary Movie 5:** VGN responses to 90° static tilt. Two sequential movie clips at ×4 playback speed showing ΔR/R0 VGN responses to 90° static tilt. First clip: pitch axis. Second clip: roll axis. Maximum intensity projection images. Single trial. Dashed line indicates VG contour.

**Supplementary Movie 6:** VGN responses to vibration stimulus. Two sequential movie clips at ×4 playback speed showing ΔR/R0 VGN responses to vibration stimulus. First clip: roll axis. Second clip: pitch axis. Maximum intensity projection images. Single trial. A VGN responds to both stimuli (arrowhead) whereas another VGN responds to only pitch vibration (arrow). Dashed line indicates VG contour.
