# Supplementary material for: Tiltable objective microscope visualizes selectivity for head motion direction and dynamics in zebrafish vestibular system
Source: Nat Commun. 2022 Dec 21;13:7622. doi: 10.1038/s41467-022-35190-9 (PMC9772181; doi:10.1038/s41467-022-35190-9)
Supplement: Supplementary file 10 — Reporting Summary [file 41467_2022_35190_MOESM10_ESM.pdf]

nature portfolio

Corresponding author(s): Masashi Tanimoto
Shin-ichi Higashijima

Last updated by author(s): Nov 3, 2022

Reporting Summary

Nature Portfolio wishes to improve the reproducibility of the work that we publish. This form provides structure for consistency and transparency in reporting. For further information on Nature Portfolio policies, see our Editorial Policies and the Editorial Policy Checklist.

Statistics

For all statistical analyses, confirm that the following items are present in the figure legend, table legend, main text, or Methods section.

n/a	Confirmed	
☐	☒	The exact sample size (*n*) for each experimental group/condition, given as a discrete number and unit of measurement
☐	☒	A statement on whether measurements were taken from distinct samples or whether the same sample was measured repeatedly
☐	☒	The statistical test(s) used AND whether they are one- or two-sided<br>*Only common tests should be described solely by name; describe more complex techniques in the Methods section.*
☒	☐	A description of all covariates tested
☒	☐	A description of any assumptions or corrections, such as tests of normality and adjustment for multiple comparisons
☐	☒	A full description of the statistical parameters including central tendency (e.g. means) or other basic estimates (e.g. regression coefficient) AND variation (e.g. standard deviation) or associated estimates of uncertainty (e.g. confidence intervals)
☐	☒	For null hypothesis testing, the test statistic (e.g. *F*, *t*, *r*) with confidence intervals, effect sizes, degrees of freedom and *P* value noted<br>*Give P values as exact values whenever suitable.*
☒	☐	For Bayesian analysis, information on the choice of priors and Markov chain Monte Carlo settings
☒	☐	For hierarchical and complex designs, identification of the appropriate level for tests and full reporting of outcomes
☒	☐	Estimates of effect sizes (e.g. Cohen's *d*, Pearson's *r*), indicating how they were calculated

Our web collection on statistics for biologists contains articles on many of the points above.

Software and code

Policy information about availability of computer code

Data collection	HC Image Live version 4.4.0.11 (Hamamatsu) and Kinesis version 1.14.18 (ThorLabs) were used for functional imaging. LAS X version 3.5.7.23225 (Leica) was used for non-functional imaging.
Data analysis	ImageJ (version 1.52a) and custom MATLAB (version 2019b) code were used to analyze images as described in the Methods section. The custom code is available at Zenodo repository, https://doi.org/10.5281/zenodo.7147882. Python (version 3.7.6) with SciPy (version 1.5.2) was used for statistical analysis.

For manuscripts utilizing custom algorithms or software that are central to the research but not yet described in published literature, software must be made available to editors and reviewers. We strongly encourage code deposition in a community repository (e.g. GitHub). See the Nature Portfolio guidelines for submitting code & software for further information.

Data

Policy information about availability of data

All manuscripts must include a data availability statement. This statement should provide the following information, where applicable:
- Accession codes, unique identifiers, or web links for publicly available datasets
- A description of any restrictions on data availability
- For clinical datasets or third party data, please ensure that the statement adheres to our policy

The datasets that support the findings of this study are available in Zenodo with the identifier doi: 10.5281/zenodo.7147882.

Human research participants

Policy information about studies involving human research participants and Sex and Gender in Research.

Reporting on sex and gender	n/a
Population characteristics	n/a
Recruitment	n/a
Ethics oversight	n/a

Note that full information on the approval of the study protocol must also be provided in the manuscript.

Field-specific reporting

Please select the one below that is the best fit for your research. If you are not sure, read the appropriate sections before making your selection.

- [x] Life sciences
- [ ] Behavioural & social sciences
- [ ] Ecological, evolutionary & environmental sciences

For a reference copy of the document with all sections, see nature.com/documents/nr-reporting-summary-flat.pdf

Life sciences study design

All studies must disclose on these points even when the disclosure is negative.

Sample size	Number of samples was as follows. For functional imaging, n=12 for utricular otolith organs (n=6 for Tg[myo6b:jGCaMP7f; myo6b:tdTomato] and n=6 for Tg[myo6b:jGCaMP7f;s100s-hs:tdTomato]); n=6 for vestibular ganglia. For non-functional imaging, n=5. No statistical methods were used to predetermine sample sizes. However, sample sizes are consistent with those reported in previous publications and are thought to be sufficient because of observed consistency between organs examined.
Data exclusions	As described in Methods section, fluorescent beads that aggregated with each other were manually excluded from data analysis (Fig. 1). In morphological analysis of photoconverted VGNs (Fig. 7), a few Kaede-expressing cells, which were not VGNs, were excluded from the analysis, and voxel position range in which fluorescence voxel data existed in all ear samples was analyzed. In Supplementary Fig. 5, VGN position range in which VGN fluorescence data existed at least one ear sample was analyzed.
Replication	For functional imaging, utricular otolith organs from 4 fish (Tg[myo6b:jGCaMP7f; myo6b:tdTomato]), those from 3 fish (Tg [myo6b:jGCaMP7f;s100s-hs:tdTomato]), and vestibular ganglia from 3 fish were independently examined. For non-functional imaging, 4 fish were independently examined. All replication attempts were successful and observed direction selectivity patterns were consistent with previously described morphological polarity patterns.
Randomization	Fish used for imaging were selected randomly. All organs that passed quality control were analyzed equally. Thus, there was no requirement for randomization.
Blinding	Blinding was not possible as fish lines and cell types were evident from the image data. Quantifications were applied equally to all the fish lines and cell types.

Reporting for specific materials, systems and methods

We require information from authors about some types of materials, experimental systems and methods used in many studies. Here, indicate whether each material, system or method listed is relevant to your study. If you are not sure if a list item applies to your research, read the appropriate section before selecting a response.

Materials & experimental systems

n/a	Involved in the study	
x		Antibodies
x		Eukaryotic cell lines
x		Palaeontology and archaeology
	x	Animals and other organisms
x		Clinical data
x		Dual use research of concern

Methods

n/a	Involved in the study	
x		ChIP-seq
x		Flow cytometry
x		MRI-based neuroimaging

Animals and other research organisms

Policy information about studies involving animals; ARRIVE guidelines recommended for reporting animal research, and Sex and Gender in Research

Laboratory animals	Transgenic zebrafish larvae at 5 days postfertilization were used.
Wild animals	No wild animals were used in this study.
Reporting on sex	Sex is not yet determined at the developmental stage of animals studied. Thus, information on sex has not been collected and sex-based analysis has not been performed.
Field-collected samples	No field-collected samples were used in this study.
Ethics oversight	Experiments were performed under the procedure #22A034 approved by the animal care and use committees of the National Institutes of Natural Sciences.

Note that full information on the approval of the study protocol must also be provided in the manuscript.
